# Differentiation and description of aromatic short grain rice landraces of eastern Indian state of Odisha based on qualitative phenotypic descriptors

**DOI:** 10.1186/s12898-016-0086-8

**Published:** 2016-08-09

**Authors:** Pritesh Sundar Roy, Rashmita Samal, Gundimeda Jwala Narasimha Rao, Sasank Sekhar Chyau Patnaik, Nitiprasad Namdeorao Jambhulkar, Ashok Patnaik, Trilochan Mohapatra

**Affiliations:** 1 Central Rice Research Institute, Cuttack, 753006 India; 2 ICAR, DARE, New Delhi, 110001 India

**Keywords:** Rice, Landraces, Aromatic short grain, Phenotypic, Trait, Characterization, Diversity

## Abstract

**Background:**

Speciality rice, in general, and aromatic rice in particular, possess enormous market potential for enhancing farm profits. However, systematic characterization of the diversity present in this natural wealth is a major pre requisite for using it in the breeding programs. This study reports qualitative phenotypic trait based characterization of 126 short grain aromatic rice genotypes, collected from different areas of the state of Odisha, India.

**Results:**

Out of the 24 descriptors employed, highest variability (8 different types) was observed for lemma-palea colour with a genetic diversity index (*He*) of 0.696. The principal component analysis reveals that the tip colour of lemma, colour of awn and colour of stigma, cumulatively explain 74 % of the total variation. The Population STRUCTURE analysis classified the population into two subpopulations which were subdivided further into four distinct groups. The western and southern districts of Odisha are endowed with maximum diversity in comparison to eastern and northern districts and at district level comparisons, Koraput and Puri districts are rich with a genetic diversity values of 0.324 and 0.303 respectively. With this set of morphological qualitative traits, based on ‘phenoprinting’, a newly proposed bar coding system, unique fingerprints of each genotype can be effectively generated that can help in easy identification of these genotypes.

**Conclusion:**

Though aromatic rices represent a tiny fraction of the total rice germplasm, a small collection of 126 land races did exhibit rich diversity for all the qualitative traits. For lemma-palea colour, eight different types were detected while for tip colour of lemma, six different types were recorded, suggesting the presence of rich variability in short grain aromatic rices that are conserved in this region. The proposed ‘phenoprinting’ can be an effective descriptor with the unique finger prints generated for each genotype and coupled with molecular (DNA) finger printing, we can discriminate and identify each and every aromatic short grain rice genotype. The proposed system not only help in conservation but also can confer IPR protection to these specialty rices.

**Electronic supplementary material:**

The online version of this article (doi:10.1186/s12898-016-0086-8) contains supplementary material, which is available to authorized users.

## Background

A large and diverse set of aromatic rice landraces are being maintained in different gene banks of India, which have been assembled by various explorations spread over several decades. Many accessions of these accessions have not yet been characterized [1]. Since landraces are thought to be the intermediate group between the wild ancestors and cultivated varieties [2], characterization of this wealth is of relevance for isolation of desirable genotypes as donors of useful traits. India has rich genetic diversity of aromatic rice landraces, majority of them having small to medium grains [3–6]. Aromatic rices constitute a small but an important sub-group (Group V) identified by Glaszmann [7] with a set of 15 isozyme markers. Other than Basmati, the indigenous short grain aromatic rices are grown in localized pockets in almost all states of the country [8, 9]. These rices possessing unique aroma, cooking and eating qualities are consumed locally as a delicacy [10, 11]. The aromatic rice varieties have evolved over thousands of years in nature and in the small farms of local farmers who select different types to suit their local cultivation practices and needs [12].

Odisha, an ancient state of India, is one of the major producer and consumer of aromatic rices. The state has its own set of aromatic short grain rices, which are being cultivated in almost all districts in different agro-climatic zones [6, 13]. These specialty rices are treated as sacred among people. Many of them like Deulabhog, Durgabhog, Krishnabhog, Prasadbhog etc. are being used in several temple duties [14]. The aromatic short grain rice landraces enriched in cooking and eating quality features hold enormous potential to be utilized as value added products, thereby providing higher economic return to small and marginal farmers [15]. Further, these native short grain aromatic rices hold significance over traditional Basmati rice for their varied and intense aroma with long retention in relatively warmer climate [3]. Characterization of these landraces is a prerequisite for understanding the extent of diversity, identification of valuable traits required for aromatic rice improvement and defining the conservation needs. Jeypore tract of Odisha is known for its biodiversity as secondary centre for origin of rice [16, 17]. Characterization of aromatic rice genotypes from Jeypore and its nearby districts can provide insights into the origin and spread of these rices. Since, rice is a self-pollinated crop and morphological description of rice plant is well established, its phenotypic descriptors could serve as potential marker for varietal identification and differentiation. Phenotypic characterization of the aromatic landraces would be a primary but essential measure leading to cataloguing of these unique high quality and diverse group, thereby strengthening their conservation and utilization strategies.

Despite that Odisha holds significance from the view point of origin and spread of rice, the status of aromatic short grain rice genotypes of this Indian state has not been studied extensively. The present study investigates structure and diversity among the aromatic short grain rice genotypes (ASGs) collected from different districts of Odisha.

## Methods

### Plant material collection and characterization

One hundred and twenty-six short grain aromatic rice lines (grains/panicles) were collected by germplasm experts and plant breeders of Central Rice Research Institute (CRRI), Cuttack from different parts of Odisha, India (Additional file 1: Table S1) during an exploration and collection programme between 2005 and 2009 conducted by the Institute. Further, the genotypes were grouped under 19 geographical districts based on their area of collection (Additional file 2: Figure S1). The genotypes were transplanted in randomised complete block design in different experimental plots of CRRI and the genetic purity of each landrace was maintained by periodic removal of ‘off types’ in four purification cycles. The experiment was conducted under controlled environmental condition with recommended dosage of fertilizers to minimize any environmental error. The purified rice genotypes were grown in 2 m × 2 m plot, from which 10 hills were sampled for characterization. Twenty-four phenotypic characters were measured at different growth stages following the guidelines of International Rice Research Institute for three consecutive years i.e. 2013–2015 [18].

### Data analysis

Data for 24 qualitative phenotypic traits were recorded on 10 random plants per accession for 3 years and their means were calculated for analysis (Table 1). The qualitative phenotypic traits were coded for the presence (1) and absence (0) of each alternate form. Diversity parameters viz., number of alleles/variables (*Na*), effective number of alleles/variables (*Ne*), Shannon Index (*I*) and unbiased Nei’s genetic diversity index (*He*) [19] were calculated using POPGENE v 1.32 [20] with 1000 permutations. The neighbour joining dendrogram based on Nei’s unbiased pairwise genetic distances among genotypes was constructed in MEGA 6 [21]. District wise genetic diversity measures were calculated using POPGENE v 1.32 and the neighbour joining tree was constructed with pairwise genetic distance measures between districts by MEGA 6. The molecular variance was analysed by hierarchical AMOVA implemented in the software GeneAlEx6 [22] based on unbiased Fst estimator as described by Weir and Cockerham [23] to estimate genetic variation among and within districts. Significance of the F-statistics was tested by non-differentiation of probability for 10,000 randomizations. The Bayesian model-based clustering analysis of the genotypes was used for determining the optimal number of genetic clusters found among rice varieties by the software STRUCTURE [24] using admix approach with 100,000 burn-in (iteration) periods followed by 100,000 Markov Chain Monte Carlo (MCMC) replicates with ten independent runs (K) ranging from 1 to 10. The ΔK based on the change in the log probability of the data between successive K values was estimated using the parameters described by Evanno and colleagues [25] with Structure Harvester v6.0 [26] and the inferred population clusters were produced by Structure Plot [27] (http://www.btismysore.in/strplot). Individuals with probability of membership >80 % were assigned to the same group, while those with <80 % probability membership in any single group were assigned to a admixed group [28]. We used a graphic presentation method based on alternate forms for the morphological descriptors, which we describe as ‘Phenoprint’. The Phenoprint was developed using Microsoft Excel 2013, in which the binary data were represented as bar(s); black bar for the presence of the variable and grey bar for the absence to provide uniqueness for each of the genotype. Different morphological characters that contributed most to the observed phenotypic variance were identified by principal component analysis (PCA) in the software SAS 9.2 [29]. The subset regression was used to identify different principal components that contributed to the total variance in the dataset.

**Table 1:** Phenotypic traits used in the present study for diversity analysis of 126 aromatic short grain rice genotypes

Trait name	Variable	Trait name	Variable
Basal leaf sheath colour BLS	Green (1)	Leaf: shape of ligule LSL	Truncate (36)
	Light purple (2)		Acute (37)
	Purple lines (3)		Split (38)
	Purple (4)	Leaf: auricles LAR	Absent (39)
Flag leaf attitude of blade FLA	Erect (5)		Present (40)
	Semi erect (6)	Spikelet: colour of stigma CSTG	White (41)
	Horizontal (7)		Yellow (42)
	Deflexed (8)		Light purple (43)
Culm angle CA	Erect (9)		Purple (44)
	Semi erect (10)	Panicle: attitude of branches PAB	Erect (45)
Stem thickness ST	Week (11)		Semierect (46)
	Medium (12)		Semierect to spreading (47)
	Strong (13)		Spreading (48)
Panicle: awns PA	Absent (14)	Collar colour CC	Pale green (49)
	Present (15)	Internode colour INC	Green (50)
Panicle: colour of awns PCA	Yellowish white (16)		Light gold (51)
	Light red (17)		Purple line (52)
	Purple (18)	Panicle secondary branching PSB	Weak (53)
	Black (19)		Strong (54)
Panicle: curvature of main axis PMA	Straight (20)	Leaf angle LAG	Erect (55)
	Dropping (21)		Semi erect (56)
Spikelet: tip colour of lemma SCL	White (22)		Horizontal (57)
	Yellowish (23)		Droopy (58)
	Brown (24)	Lemma palea colour LPC	Straw (59)
	Red (25)		Brown Furrow (60)
	Purple (26)		Brown (61)
	Black (27)		Red (62)
Panicle exertion PE	Partly exserted (28)		Purple (63)
	Exserted (29)		Purple Furrow (64)
	Well exserted (30)		Black (65)
Leaf intensity of blade colour LI	Medium (31)		Brown Spot (66)
Leaf: anthocyanin colour LA	Absent (32)	Grain type GT	Short bold (67)
Leaf: pubescence of blade surface LPB	Medium (33)		Medium bold (68)
Leaf: ligule LL	Present (34)		Medium slender (69)
Leaf: colour of ligule CL	Green (35)		Medium long (70)

Variables only recorded in the present set of germplasm are given. Numbers in parenthesis in the last column represents order of variable for individual trait in the phenoprint of Fig. 3b

## Results

### Phenotypic trait diversity

**Fig. 1:**
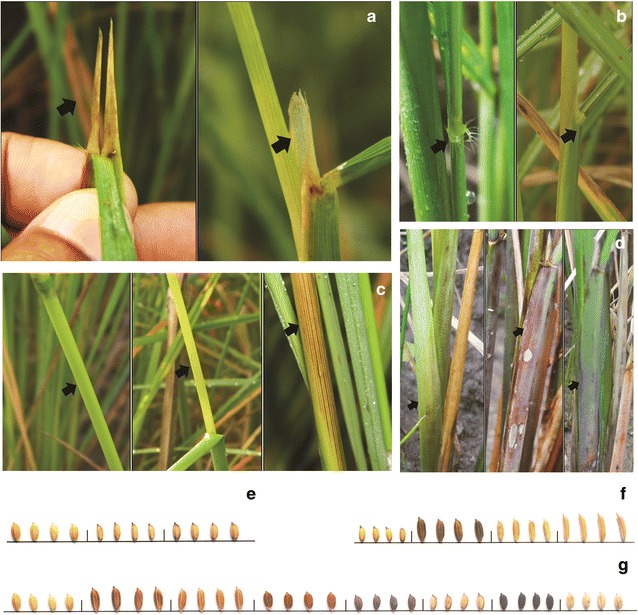
Variations in aromatic short grain rice genotypes for different phenotypic characters. **a** Shape of ligule (L–R: *split*, *acute*); **b** leaf auricle (L–R: *present*, *absent*); **c** internode colour (L–R: *green*, *light gold*, *purple line*); **d** basal leaf sheath colour (L–R: *green*, *light purple*, *purple line*, *purple*); **e** tip colour of lemma (L–R: *white*, *purple*, *black*); **f** grain type (L–R: *short* *bold*, *medium* *bold*, *medium* *slender*, *medium* *long*); **g** lemma-palea colour (*straw*, *brown* *furrow*, *brown*, *red*, *purple*, *purple* *furrow*, *black*, *brown* *spot*)

Eighteen (75 %) out of 24 phenotypic traits showed variation among the 126 indigenous short grain aromatic rice genotypes, whereas no variation was observed for the traits like intensity of blade colour (LI), leaf anthocyanin colour (LA), pubescence of blade surface (LPB), leaf ligule (LL), colour of ligule (CL) and collar colour (CC) (Table 1). Phenotypic variation for some of the traits are given in Fig. 1. The number of variables per individual trait ranged from 1 (traits having no variation) to a maximum of 8 for lemma-palea colour with a total number of 70 variables with an average of 2.92 per trait. The genetic diversity index (*He*) for different traits ranged from 0.016 (lowest) to 0.696 (highest) for culm angle and lemma-palea colour, respectively with an average of 0.286. The number of effective variables (*Ne*) ranged from 1 to 3.292 with an average of 2.958 (Table 2). Further, frequency distribution of the phenotypic traits against the studied genotypes indicated unequal distribution of variables under each trait (Additional file 2: Figure S2). Among the 126 genotypes, presence of auricles (99 %), semi erect culm angle (99 %), split ligule (95 %), absence of awn (91 %), drooping type panicles (91 %), semi erect attitude of panicle branches (86 %), well exserted panicle (76 %), white colour stigma (74 %), light gold colour internodes (71 %), yellowish white colour of awns (64 %), branching of panicles weak (63 %), medium stem thickness (55 %), green basal leaf sheath colour (51 %), lemma having purple tip (45 %) and erect flag leaf attitude of blade (44 %) were predominant. A comparatively equal frequency of distribution for short bold (47 %) and medium bold (51 %) grain type were recorded for the genotypes. In case of lemma-palea colour that showed maximum variation with eight alternative variables, straw colour was most predominant (50 %) followed by purple furrow (14 %), brown (11 %), purple and brown furrow (10 % each), black and red (2 % each) and brown spot (1 %).

**Table 2:** Diversity parameters of phenotypic traits

Phenotypic characters	*Na*	*Ne*	*He*	*I*
Basal leaf sheath colour	4	2.590	0.614	1.098
Flag leaf attitude of blade	4	2.886	0.653	1.140
Culm angle	2	1.016	0.016	0.046
Stem thickness	3	2.477	0.596	0.999
Panicle: awns	2	1.190	0.159	0.296
Panicle: colour of awns	4	1.196	0.164	0.387
Panicle: curvature of main axis	2	1.190	0.159	0.296
Spikelet: tip colour of lemma	6	3.065	0.674	1.273
Panicle exertion	3	1.609	0.379	0.656
Leaf intensity of blade colour	1	1.000	0.000	0.000
Leaf: anthocyanin colour	1	1.000	0.000	0.000
Leaf: pubescence of blade surface	1	1.000	0.000	0.000
Leaf: ligule	1	1.000	0.000	0.000
Leaf: colour of ligule	1	1.000	0.000	0.000
Leaf: shape of ligule	3	1.101	0.091	0.213
Leaf: auricles	2	1.016	0.016	0.046
Spikelet: colour of stigma	4	1.751	0.429	0.841
Panicle: attitude of branches	4	1.335	0.251	0.490
Collar colour	1	1.000	0.000	0.000
Internode colour	3	1.766	0.434	0.739
Panicle secondary branching	2	1.864	0.464	0.656
Leaf angle	4	2.161	0.537	0.859
Lemma-palea colour	8	3.292	0.696	1.518
Grain type	4	2.094	0.522	0.804
Mean	2.92	2.958	0.286	0.515
St. Dev.	1.780	1.781	0.262	0.474

*Na* number of variables, *Ne* effective number of variables, *He* Nei’s genetic diversity, *I* Shannon diversity index

**Fig. 2:**
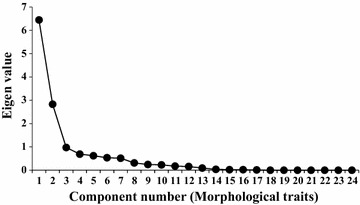
Scree plot of principal component analysis (PCA) showing the number of morphological traits and their importance for grouping of aromatic short grain rice landraces

The principal component analysis (PCA) showed that 3 of the 24 morphological traits were the most important components for explaining the grouping of genotypes (Fig. 2). Principal component analysis showed that, first, second, third, fourth, and fifth principal components accounted for 46, 20, 7, 5 and 4 % of the total variance, respectively. The first three principal components together explained a cumulative 74 % of the total variance. Further, the subset regression analysis could identify tip colour of lemma, colour of awn and colour of stigma as the three most important traits. Grain type, flag leaf attitude of blade and lemma-palea colour were identified to be secondary traits to explain the maximum available phenotypic variability within the set of studied genotypes. However, ten (tip colour of lemma, colour of awn, colour of stigma, grain type, flag leaf attitude, lemma palea colour, attitude of panicle branches, basal leaf sheath colour, leaf angle and internode colour) out of 24 morphological traits could adequately explain the total variation existing in these short grain aromatic rice genotypes (Table 2).

### Genetic relationship and population structure

**Fig. 3:**
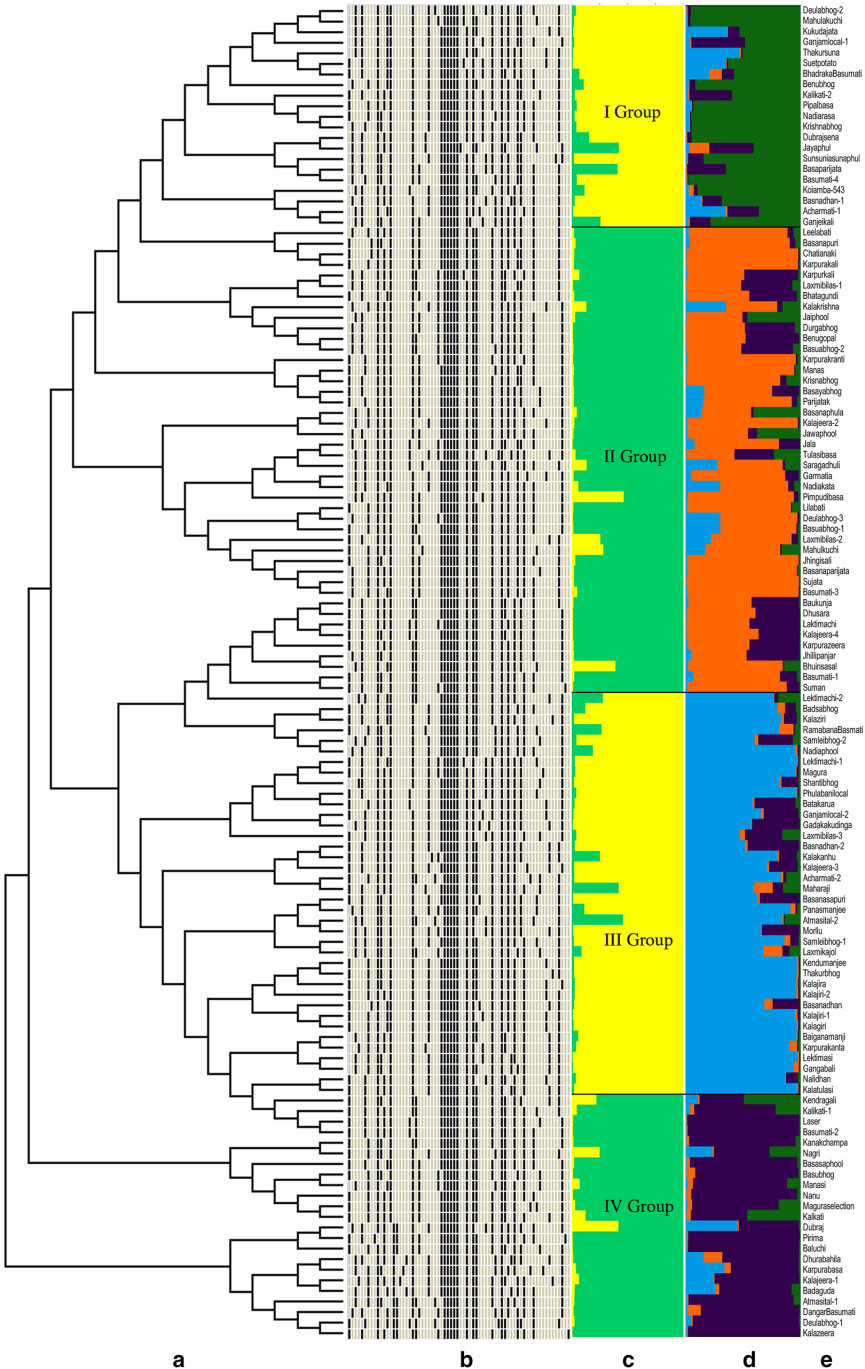
Genetic diversity, population structure and phenoprint representation of 126 indigenous aromatic short grain rice genotypes based on 71 variables for 24 phenotypic traits. **a** Neighbour-joining dendrogram based on Nei’s genetic distance showing genetic relationship among indigenous aromatic short grain rice genotypes; **b** phenoprint illustration of indigenous aromatic short grain rice genotypes (*Each row* corresponds to a genotype and *each column* represents phenoprinting pattern of a variable across the genotype); **c** population structure for indigenous aromatic short grain rice genotypes estimated by the STRUCTURE programme at K = 2; **d** population STRUCTURE at K = 4; **e** names of the corresponding genotypes

The pairwise Nei’s genetic distance was calculated to understand the genetic diversity and relatedness among the 126 short grain aromatic rice genotypes, which ranged from 0.426 (lowest) to 0.875 (highest) with an average of 0.347. The neighbour-joining dendrogram constructed based on Nei’s unbiased pairwise genetic distance grouped the rice genotypes into 2 major clusters (Fig. 3a), of which, Cluster I consisted of 115 genotypes while the rest 11 genotypes grouped in Cluster II. Since, the genotypes differed from each other for at least one or more morphological characters, no duplicates with identical features were detected in the entire set of germplasm.

Bayesian analysis of population structure using the model-based approach with the admixture model proposed by Falush and colleagues [30] provided support for the existence of population structure. The maximum log likelihood (84.76) was observed for K = 2 (Additional file 2: Figure S3). The genotypes were differentiated into two subpopulations i.e. SP 1 with a membership percentage of 54.8 % while SP 2 with 45.2 %. The fixation index (Fst) values of subpopulations were 0.1415 and 0.2794 for SP 1 and 2, respectively, while the pairwise allele frequency divergence value between the subpopulations was 0.0489 (Additional file 1: Table S2), indicating the existence of moderate population structure in these genotypes. Further, 8 and 7 genotypes were identified to be admix type having a membership value of <80 % in the subpopulations SP1 and SP2, respectively. Moreover, at K = 4, the 126 short grain aromatic rice genotypes could be further differentiated into four groups. (Fig. 3c, d). Forty-four genotypes were clustered in Group II followed by 38 in Group III, 23 in Group IV and 21 genotypes in Group I. Relationship among the genotypes obtained from the STRUCTURE based clustering was in line with the NJ dendrogram except a few deviations.

### Phenoprint development

The phenoprints of the aromatic short grain rice genotypes were developed based on 70 variables of the 24 morphological characters (Fig. 3b). The phenoprints uniquely identified each landrace and differentiated them from each other for at least one morphological character. Moreover, it provided a clear depiction of variation for identification of genotypes based on their morphological attributes. For instance, when phenotypic trait of different genotypes were compared, their phenoprints depicted Atmasital-1 having purple lines for basal leaf sheath colour, while light purple in Dangar Basumati, and green in Deulabhog-1. The probability of identical match by chance based on all the polymorphic traits was 2.9 × 10^−4^, with a differentiation possibility of 10^4^ genotypes. This suggested that the phenoprints can be used as identifying tags for the ASGs (aromatic short grain rices). Different accessions with the same name like Deulabhog, Lektimachi, Acharmati, Atmasital, Ganjamlocal, Kalikati, Basnadhan, Basumati, Laxmibilas, Samleibhog, Basuabhog and Kalajeera collected from different geographical areas differed from each other for more than one qualitative morphological characters. For instance, the two accessions of Samleibhog (collected from Sundargarh area) were different for the characters like panicle attitude of branches, panicle secondary branching, lemma-palea colour and grain type. The three accessions of Deulabhog (collected from Puri and Koraput) showed variation for a maximum of 14 out of 24 morphological traits.

Further, we performed a heuristic search and identified 16 genotypes (12.7 %) i.e. Deulabhog-1 (Puri), Lektimasi (Malkangiri), Jayaphul (Sundargarh), Karpurabasa (Koraput), Lektimachi-1 (Malkangiri), Pirima (Koraput), Kalajeera-1 (Mayurbhanj), Gadakakudinga (Phulbani), Garmatia (Puri), Saragadhuli (Cuttack), Ganjeikali (Dhenkanal), Kalazeera (Dhenkanal), Basanadhan (Koraput), Dhurabahila (Koraput), Sunsuniasunaphul (Deogarh) and Kalakanhu (Bolangir), which represented the maximum available phenotypic variation (more than 98 %) for the 24 phenotypic traits recorded. Higher numbers of genotypes (6) in this group were recorded from Koraput and Malkangiri region. The average Nei’s genetic diversity value of 0.245 detected for this group of genotypes was comparable with genetic diversity value of the complete set (0.286).

### Differentiation of aromatic short grain rice genotypes based on geographical districts of collection

**Fig. 4:**
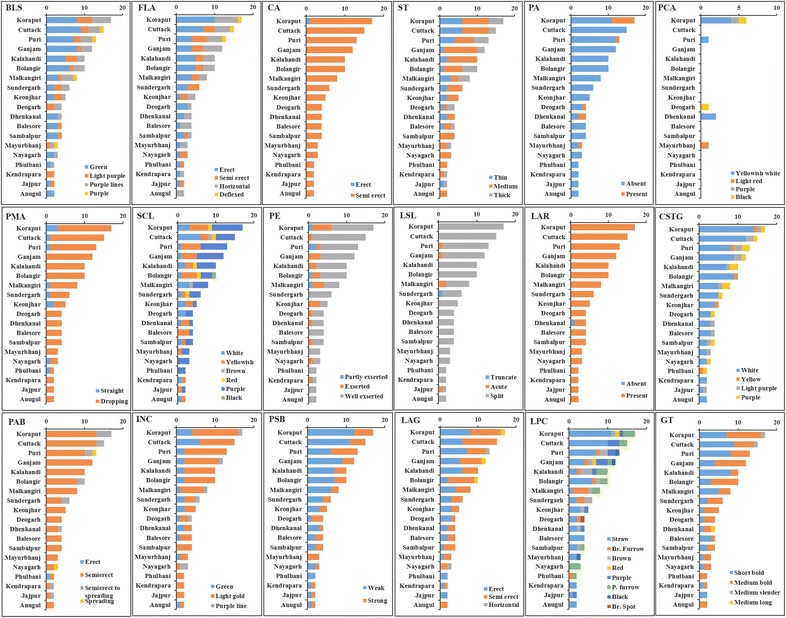
Distribution of qualitative phenotypic traits in 19 geographical districts of collection

**Fig. 5:**
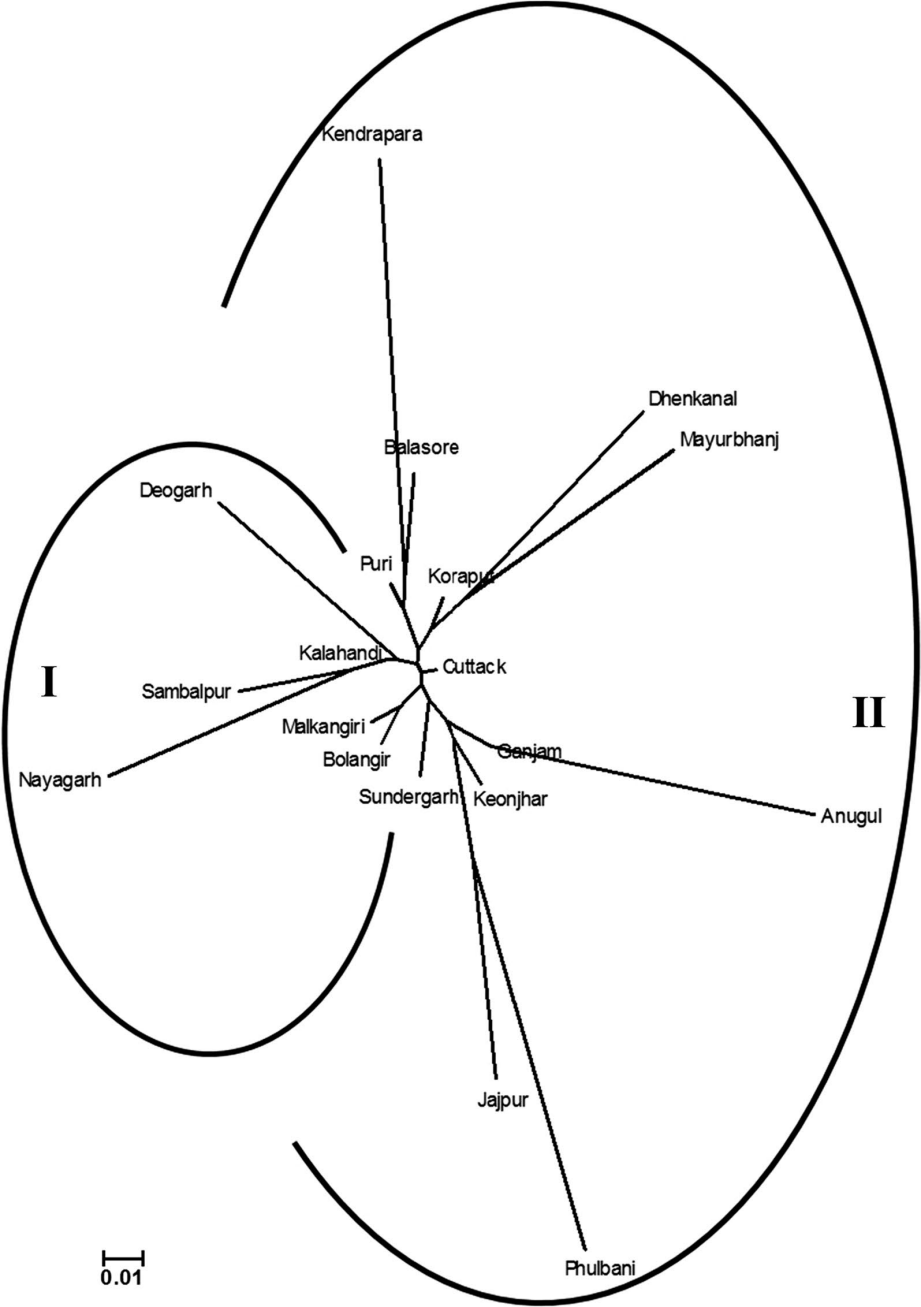
Unrooted neighbour-joining *tree* showing genetic relationship among 19 geographical districts based on phenotypic of aromatic short grain rice landraces

The aromatic short grain rice genotypes collected and screened in our study were represented by 19 different districts of Odisha. We grouped the genotypes based on these 19 districts to address the phenotypic variability within a particular district. In our collection, maximum (17) number of genotypes were from Koraput followed by Cuttack (15) and Ganjam (12) whereas, only limited number of genotypes (2 each) were included from Anugul, Jajpur, Kendrapara and Phulbani districts. We could detect a non-uniform but wide distribution of phenotypic characters like basal leaf sheath colour, flag leaf attitude of blade, tip colour of lemma, colour of stigma, lemma-palea colour and grain type in all the districts (Fig. 4). However, a comparatively uniform but narrow distribution of phenotypic characters was recorded for culm angle (exception of Koraput) and leaf auricles (exception of Dhenkanal). In case of panicle secondary branching, all the district except Phulbani contained accession with both the alternate forms. A high level of variation was observed in Koraput and Puri districts, whereas it was significantly low in Anugul, Jajpur, Kendrapara and Phulbani districts. Genetic diversity parameters revealed that effective number of variables was high for Puri district (1.703) as compared to Anugul (1.167) (Table 3). Similarly, the highest value for Nei’s genetic diversity was recorded in the germplasm set of Koraput (0.324) followed by Puri (0.303), Anugul having the lowest (0.083). The values for Shannon’s information index were in line with the genetic diversity values. However, when we calculated the polymorphism percentage of genotypes for the 24 qualitative phenotypic traits, greater part of the variation was observed in Koraput district (67.61 %) followed by Puri (64.79 %), while, the genotypes from Anugul district represented a minimum variation of 8.45 %. The pair-wise comparisons of Nei’s unbiased genetic distance between the 19 districts estimated based on the qualitative morphological traits ranged from 0.370 (between Phulbani and Kendrapara) to 0.013 (between Kalahandi and Cuttack) with an average distance value of 0.097 (Additional file 1: Table S3). The neighbour-joining tree constructed based on Nei’s genetic distance grouped the 19 geographical regions into two major clusters (Fig. 5). The six districts i.e. Sambalpur, Kalahandi, Deogarh, Malkangiri, Bolangir and Sundargarh, which are close to the border of nearby state, Chhattisgarh on the western side of Odisha, were grouped in one cluster (I) with only exception of Nayagarh. The rest of the districts were grouped in cluster II. Genotypes from Puri, Kendrapara, Balasore, Koraput, Mayurbhanj, Dhenkanal, Ganjam, Anugul, Keonjhar, Jajpur and Phulbani showed close relationship among them. Further, analysis of molecular variance (AMOVA) based on geographical districts indicated that on an average, 92 % variation exists within districts and 8 % variation was observed among the district (Table 4).

**Table 3:** Diversity parameters of 126 aromatic short grain rice genotypes based on 19 geographical districts of collection

District	Co-ordinates	No. of genotypes	*Ne*	*He*	*I*	*% P*
Anugul	20°47′50″N 85°1′26″E	2	1.167	0.083	0.116	8.45
Balasore	21.49°N 86.93°E	4	1.411	0.219	0.323	26.76
Bolangir	20.72°N 83.48°E	10	1.564	0.251	0.420	47.89
Cuttack	20.27°N 85.52°E	15	1.568	0.255	0.423	52.11
Deogarh	21.53°N 84.73°E	4	1.356	0.182	0.280	40.85
Dhenkanal	20.67°N 85.6°E	4	1.569	0.266	0.406	46.48
Ganjam	19.38°N 85.07°E	12	1.542	0.234	0.401	50.70
Jajpur	20.85°N 86.333°E	2	1.208	0.104	0.144	25.35
Kalahandi	20.083°N 83.2°E	10	1.492	0.225	0.373	46.48
Kendrapara	20.525°N 86.475°E	2	1.292	0.146	0.202	22.54
Keonjhar	21.63°N 85.58°E	5	1.542	0.230	0.362	39.44
Koraput	18.8083°N 82.7083°E	17	1.780	0.324	0.546	67.61
Malkangiri	18.35°N 81.90°E	8	1.521	0.225	0.379	50.70
Mayurbhanj	21.933°N 86.733°E	3	1.500	0.241	0.357	32.39
Nayagarh	20.116°N 85.01°E	3	1.383	0.194	0.284	28.17
Phulbani	20.47°N 84.23°E	2	1.208	0.104	0.144	16.90
Puri	20.47°N 84.23°E	13	1.703	0.303	0.517	64.79
Sambalpur	19.48°N 85.49°E	4	1.464	0.240	0.361	30.99
Sundargarh	21.47°N 83.97°E	6	1.530	0.241	0.384	43.66

*Ne* average effective number of variables, *He* average Nei’s genetic diversity, *I* average Shannon’s information index, *%* *P* percent polymorphism

**Table 4:** Analysis of molecular variance for 126 short grain aromatic rice genotypes from 19 geographical districts

Source	df	SS	MS	*F*_ST_	EV	%
Among population	18	120.880	6.716	0.079	0.274	8
Within population	233	742.723	3.188		3.188	92
Total	251	863.603	–		3.461	100

*df* degree of freedom, *SS* sum of squares, *MS* mean squares, *EV* estimated variance (p > 0.001)

## Discussion

Assessment of genetic variation in the short grain aromatic rices of a particular region has implications for conservation of these neglected heritage resource and unlocking genes controlling valuable traits for their utilization in rice improvement [31]. Further, the state of Odisha being a major producer of rice in India and having a large assembly of indigenous rice genotypes [13], there was an imperative need to characterize them systematically.

### Overall phenotypic variation

In this study, we could identify eighteen, out of 24 phenotypic traits which showed significant variation in the ASG rice collection. A total of 70 variables were detected by 24 phenotypic traits in our study, which is significantly higher than that in aromatic rice germplasm of Madhya Pradesh and Chhattisgarh of India [32], in upland rice collection of Japan [33], in aromatic rice collection from Maharashtra and Karnataka of India [34], in aromatic short grain rice genotypes of eastern India [35] and in indigenous rice landraces of Chhattisgarh state of India [36]. Highest genetic diversity value of 0.696 was contributed by lemma-palea colour, where 8 different forms were identified which is higher than the reports of Sarawgi and colleagues [37], who reported 5 types in 782 rice germplasm collection. Since lemma and palea are sepal equivalent [38], genetic base of lemma-palea colour differentiation could be utilized as a potential tool to understand the evolutionary aspects of these native genotypes. Lemma-palea pigmentation is the character of wild rice and straw hull is similar to *indica* and black hull is similar to that in *aus* groups [39]. *Black husk4 (Bh4)* gene, encoding an amino acid transporter, fine mapped on chromosome 4, controls the black husk in *O. rufipogon* and *O. nivara*. Zhu and colleagues [40] reported that transition from black husk in wild rice to straw husk in cultivated rice is due to a functional deletion of 22 *bp* in exon 3 of *bh4* variant. The intermediate types, which were observed for this trait, could be useful to establish a lineage between aromatic rice landraces and their wild progenitors to understand their domestication process. Further, red and black husked rices having nutritive and medicinal properties, are more resistant to storage insect and pests as compared to brown husked rices [41]. Ray and colleagues [42] reported that aromatic short grain rices vary for their grain characters, which was observed in our study also, where 4 different grain types were identified. Similar to our results, high level of variation was reported for grain shape in Brazilian and Pakistan rice collections [33, 43].

In our study, genotypes having same name collected from different districts (both nearby and far apart) showed variation for one or more traits. This observation is interesting. This could be due to the naming practice being followed by traditional communities for the ASGs. Since they are offered to Gods and Goddesses in the temples, the different material in different locations might be given similar/identical names. For example Deula Bhog (Deula meaning temple; Bhog meaning offering) samples collected from Puri and Koraput districts differ for basal leaf sheath colour, flag leaf attitude of blade stem thickness, panicles characteristics, tip colour of lemma, panicle exertion, shape of ligule, colour of stigma, internode colour, leaf angle and grain type. Similarly, Laxmibilas collected from Sambalpur and Deogarh districts differ for basal leaf sheath colour, flag leaf attitude of blade, tip colour of lemma, panicle exertion, colour of stigma, internode colour, panicle secondary branching, leaf angle, lemma-palea colour and grain type. The other reason of this situation arising could be introduction of the same landrace in different locations followed by selection by local growers or farmers leading to fixation of different genotypic constitution at different locations. It has been demonstrated by Kohli and colleagues [44] using molecular marker and other studies that the landraces do possess composite genetic structure and selection exercised on the base landrace is expected to result in genetically different purelines. However, what remains common in the developed lines is the target trait i.e. short grain and aroma. In our earlier report also we have detected different grades of aroma (mild/moderate/strong) in these genotypes having similar name [45]. Similarly, Bisne and Sarawgi [46] have found variation for leaf blade colour, lemma-palea colour, apiculus colour and lemma-palea pubescence in Badshah Bhog lines. Genetic variability in Indian scented rice accessions of Hansraj were also reported by Raghunathachari and colleagues [47] by using RAPD markers.

In 6 traits (leaf intensity of blade colour, leaf anthocyanin colour, leaf pubescence of blade surface, leaf ligule, leaf colour of ligule and collar colour), we could not detect any variation for the set of aromatic rice genotypes used in this study. Of the rest 6 traits, 4 related to colour of blade, leaf, collar and ligule which was green in all the genotypes. Since anthocyanin pigmentation in different parts of rice plant is due to allelic variation and complex organization of gene(s) [48], this uniformity for colour of the four traits could be due to pleiotropic effect which has remained unchanged during the process of domestication and selection by farmers [49].

The average genetic diversity value recorded in this study was relatively low (*He* = 0.286). Similar type of result was also observed in the all India collection of aromatic rice landraces [50]. However, the genetic diversity value was comparatively high than the rice landraces of Nepal [51]. The Shannon diversity index of 0.515 detected in our germplasm set is comparatively high than the rice landraces of Santhal Paraganas of Jharkhand state, India [52]. We could identify six traits i.e. tip colour of lemma, colour of awn, grain type, flag leaf attitude of blade, lemma-palea colour and colour of stigma as the most important traits to explain the variation and these traits in combination could be utilized as key for differentiation of short grain rice genotypes at field level.

Bayesian analysis of STRUCTURE using the model-based approach suggested the presence of two optimum populations i.e. SP1 and SP2 (with a K value of 2) in these natural heritages. Choudhury and colleagues [53] also inferred 2 subpopulations in the Eastern Himalayan and Northeast Indian indigenous rice with a set of seven microsatellite markers. Similarly, 3 subpopulations were detected in Indian rice core set with molecular markers, which reveals presence of moderate population structure in the rice genotypes of India [54]. A total of 15 genotypes were determined to be admixture type, which might be due to intraspecific crosses between genotypes of SP1 and SP2 [55]. Further, the two identified populations were divided into four different groups similar to the results of Laido and colleagues [56], where similar type of groups were identified in two STRUCTURE populations in the tetraploid wheat genotypes with SSR and DArT markers.

The aromatic rice collection was found to be a reserve of many useful agronomic traits. Erect flag leaf is one of the important features and related to high yielding ability [57]. In our collection, 44 % of genotypes had erect flag leaf and these could be potential donors of the trait in high yielding aromatic rice breeding programmes. Tall plant type is prone to lodging, unresponsive to fertilizer application and hence has low yielding ability [3]. In our study, 25 genotypes were identified with thick/strong stem, which could be deployed to achieve lodging resistance under higher dose of fertilizers [58]. Similarly, 74 % of the genotypes identified with well exserted panicle type could be utilized for introgession of this trait into high yielding backgrounds. Further, 37 % of the genotypes having strong panicle secondary branching, which is another important trait related to yield, could be utilized in combination with other yield traits to overcome the low yield potential of aromatic rices [59].

Trait specific characterization of these lines would help in identifying novel genes/alleles to breed improved disease resistant quality rices. This is highly relevant in the context that in the past few decades, significant decline in cultivation and huge erosion of short grain aromatic rice germplasm in Odisha has been a major concern [60]. Artificial selection pressure and promotion of high yielding varieties and hybrids over years has led to reduced genetic base and increased genetic vulnerability in traditional rice germplasm, emphasizing the conservation, characterization and documentation of native aromatic genotypes before extinction [61–63]. The present study that describes variation in ASGs is a relevant step in this direction.

### Phenoprint of aromatic short grain rice genotypes

Bar coding tools have proven to be significantly useful for varietal differentiation and their identification, almost all of which are based on DNA markers. After the establishment of a protocol for DNA barcoding of all land plants [64], this technology has been extensively used by several researchers for barcoding of many edible plants [65–70]. However, potential application of morphology based characterization cannot be ignored, since it is having useful implication to varietal discrimination at field and farm level [71]. And hence, in this study, we have characterized 126 aromatic short grain rice genotypes of Odisha, India based on qualitative phenotypic descriptors and proposed a unique bar coding approach, named ‘phenoprinting’, for varietal description. Once established, phenoprint could be utilized for rice and other crops as well. Given that the probability of identity based on all the polymorphic traits was low, this approach would be of use in varietal identification, farmers’ rights protection and intellectual property rights issues. Since, the phenoprint deals with morphological characters, it could be generated with minimal financial inputs. The phenoprint in our study that could clearly differentiate the genotypes will be a useful guide for visual comparison of additional aromatic short grain genotypes. In fact, the traits having no variation could be easily distinguishable and the traits with maximum variability could also be precisely identified. This approach provided uniqueness to each of the lines tested, providing robustness for trait specific varietal identification. Further, the 16 genotypes identified in our study, containing more than 98 % of the variables for phenotypic traits provided the key, which could be utilized for description of ASGs and also in the rice improvement programme to overcome the genetic bottleneck in years to come.

### Phenotypic diversity in different districts

The tribal dominated Koraput is endowed with diverse and valuable flora and fauna [72]. Further, Koraput region has been recently declared as ‘Globally important agricultural heritage systems’ by the Food and Agricultural Organization of the United Nations (FAO) [73]. Our findings, revealing the highest polymorphism percentage of more than 67 % and greater diverse group of aromatic rices in Koraput as compared to other areas, justifies the National and International attention towards Koraput for various biodiversity projects. The genetic diversity parameters were similar between Koraput and Puri but were significantly higher than the other districts. Detection of high genetic diversity in the germplasm set of Puri was interesting. Puri district of Odisha is renowned for the temple of Lord Jagannath where the famous car festival takes place annually. Besides, it also houses several other temples, monasteries and holy ashrams where aromatic rices are used in different temple rituals. Hence, import of aromatic rices from other parts of the state resulting in an assembly of a diverse group of genotypes in this district is possible. Further, 8 % of the total variation observed among districts suggested that each district have their unique group of aromatic short grain rices.

The neighbour joining tree based on pairwise genetic distance estimates, grouped the six districts of western Odisha in one group. Since, western Odisha, Jharkhand and Chhattisgarh are recognized as the centre of origin of *aus* ecotypes of rice [74], close relationship among the districts of western Odisha reflects evolution of short grain rice landraces in this region. In fact, critical observations showed that number of aromatic short grain rice genotypes and their genetic diversity is comparatively on higher side in western and southern Odisha than eastern and northern Odisha. Fuller [75, 76] reported an independent domestication of rice—Neolithic in Ganga Valley and the western Odisha with archaeo-botanical evidences. Considering Koraput in western Odisha as the secondary centre of origin for rice, greater diversity in Koraput and adjoining region is expected.

## Conclusion

Native aromatic rice landraces that are highly preferred by consumers needs to be characterized which can help in varietal diagnostics purpose and their conservation [77]. Here, we have characterised native short grain aromatic genotypes of Odisha (India), one of the major consumer and producer of these native rices, based on qualitative phenotypic descriptors. For the trait lemma-palea colour, a highest of 8 different forms were detected followed by tip colour of lemma where 6 variables were identified. Tip colour of lemma, colour of awn and colour of stigma were the primary determinants for explaining the existing variation in this group of aromatic rice genotypes. The 126 genotypes were broadly grouped into 2 sub populations and 4 distinct groups by population STRUCTURE analysis, revealing the existence of moderate population structure within them. But, we did not detect any duplicates in our set. Further, western and southern districts of Odisha had maximum diverse aromatic short grain rice genotypes as compared to eastern and northern districts. Moreover, the proposed phenoprinting approach, discriminating the aromatic short grain rices based on qualitative phenotypic descriptors, could provide unique identification and description to the genotypes. Since, genetic erosion over years is a major threat to aromatic rice improvement, genetic differentiation and phenotypic description study may help in preservation of this group of indigenous short grain aromatic rices.

## Additional files

10.1186/s12898-016-0086-8 Aromatic short grain rice genotypes used in the present study and region of their collection. **Table S2.** Population statistics of the estimated clusters. **SP 1* and *SP 2* are estimated subpopulations. **Table S3.** Pair-wise Nei’s unbiased genetic distance of 19 geographical districts.
10.1186/s12898-016-0086-8 Sampling sites in nineteen districts of Odisha (India) and information on population structure. **Figure S2.** Frequency distribution of phenotypic traits among 126 short grain aromatic rice landraces. **Figure S3.** The relationship between ΔK and K showing the highest peak at K = 2.

## References

[1] Rana JC, Negi KS, Wani SA, Saxena S, Pradheep K, Pareek SK, Sofi PA (2009). Genetic resources of rice in the Western Himalayan region of India: current status. Genet Resour Crop Evol.

[2] Pusadee T, Jamjod S, Chiang YC, Rerkasem B, Schaal BA (2009). Genetic structure and isolation by distance in a landrace of Thai rice. Proc Nat Acad Sci USA.

[3] Singh RK, Gautam PL, Saxena S, Singh S, Singh RK, Singh US, Khush GS (2000). Scented rice germplasm: conservation, evaluation and utilization. Aromatic rices.

[4] Roy JK, De RN, Ghorai DP, Panda A (1983). Collection and evaluation of genetic resources of rice in India. Phytobreedon.

[5] Dikshit N, Malik SS, Mohapatra P (1992). Seed protein variability in scented rice. Oryza.

[6] Malik SS, Dikshit N, Das AB, Lodh SB (1994). Studies on morphological and physio- chemical properties of local scented rice. Oryza.

[7] Glaszmann JC (1987). Isozymes and classification of Asian rice varieties. Theor Appl Genet.

[8] Shobha Rani N, Singh RK, Singh RK, Singh US (2003). Efforts on aromatic rice improvement in India. A treatise on the scented rices of India.

[9] Shobha Rani N, Krishnaiah K. Current status and future prospects for improvement of aromatic rices in India, In: Specialty rices of the world: Breeding, production and marketing, New York: Science Publishers, Inc.; 2001. p. 49–78.

[10] Bhattacharjee P, Singhal RS, Kulkarni PR (2002). Basmati rice: a review. Int J Food Sci Tech.

[11] Ahuja SC, Pawar DVS, Ahuja U, Gupta KR (1995). Basmati rice—the scented pearl.

[12] Pachauri V, Singh MK, Singh AK, Singh S, Shakeel NA, Singh VP, Singh NK (2010). Origin and genetic diversity of aromatic rice varieties, molecular breeding and chemical and genetic basis of rice aroma. J Plant Biochem Biotechnol.

[13] Das SR (2012). Rice in Odisha. IRRI Technical Bulletin No. 16.

[14] Gangadharan C, Jaiswal PL (1985). Breeding. Rice research in India.

[15] DelCruz N, Khush GS, Singh RK, Singh US, Khush GS (2000). Rice grain quality evaluation procedures. Aromatic rices.

[16] Ramaiah K, Ghose RLM (1951). Origin and distribution of cultivated plants of South Asian rice. Indian J Genet Plant Breed.

[17] Arunachalam VA, Chaudhury SS, Sarangi SK, Ray T, Mohanty BP, Nambi VA, Mishra S (2006). Rising on rice: The story of Jeypore.

[18] IBPGR-IRRI (1980). Descriptors for rice (*Oryza sativa* L.). IBPGR-IRRI Rice Advisory Committee.

[19] Nei M (1973). Analysis of gene diversity in subdivided populations. Proc Nat Acad Sci USA.

[20] Yeh FC, Yang RC, Boyle TBJ (1999). POPGENE, version 1.32 Microsoft window-based freeware for population genetic analysis.

[21] Tamura K, Stecher G, Peterson D, Filipski A, Kumar S (2013). MEGA6: molecular evolutionary genetics analysis version 6.0. Mol Biol Evol.

[22] Peakall R, Smouse PE (2006). Genalex 6: genetic analysis in Excel. Population genetic software for teaching and research. Mol Ecol Notes.

[23] Weir BS, Cockerham CC (1984). Estimating F-statistics for the analysis of population structure. Evolution.

[24] Pritchard JK, Stephens M, Donnelly P (2000). Inference of population structure using multilocus genotype data. Genetics.

[25] Evanno G, Regnaut S, Goudet J (2005). Detecting the number of clusters of individuals using the software STRUCTURE: a simulation study. Mol Ecol.

[26] Earl DA, von Holdt BM (2012). STRUCTURE harvester: a website and program for visualizing STRUCTURE output and implementing the Evanno method. Conserv Genet Resour.

[27] Ramasamy RK, Ramasamy S, Bindroo BB, Naik VG (2014). STRUCTURE plot: a program for drawing elegant STRUCTURE bar plots in user friendly interface. Springer Plus.

[28] Zhang P, Li J, Li X, Liu X, Zhao X, Lu Y (2011). Population structure and genetic diversity in a rice core collection (*Oryza sativa* L.) investigated with SSR markers. PLoS ONE.

[29] SAS Institute (2010). SAS/STAT Version 9.2.

[30] Falush D, Stephens M, Pritchard JK (2003). Inference of population structure using multilocus genotype data: linked loci and correlated allele frequencies. Genetics.

[31] Rabbani MA, Pervaiz ZH, Masood MS (2008). Genetic diversity analysis of traditional and improved cultivars of Pakistani rice (*Oryza sativa* L.) using RAPD markers. Electron. J Biotechnol.

[32] Parikh M, Motiramani NK, Rastogi NK, Sharma B (2012). Agro-morphological characterization and assessment of variability in aromatic rice germplasm. Bangladesh J Agric Res.

[33] Nascimento WF, Silva EF, Veasey EA (2011). Agro-morphological characterization of upland rice accessions. Sci Agric.

[34] Mathure S, Shaikh A, Renuka N, Wakte K, Jawali N, Thengane R, Nadaf A (2011). Characterisation of aromatic rice (*Oryza sativa* L.) germplasm and correlation between their agronomic and quality traits. Euphytica.

[35] Subudhi HN, Swain D, Samantaray S, Singh ON (2012). Collection and agromorphological characterization of aromatic short grain rice in eastern India. Afr J Agric Res.

[36] Tandekar K, Koshta N (2014). To Study the agro morphological variation and genetic variability in rice germplasm. Middle East J Sci Res.

[37] Sarawgi AK, Subba Rao LV, Parikh M, Sharma B, Ojha GC (2013). Assessment of variability of Rice (*Oryza sativa* L.) germplasm using agro-morphological characterization. J Rice Res.

[38] Lombardo F, Yoshida H (2015). Interpreting lemma and palea homologies: a point of view from rice floral mutants. Front Plant Sci.

[39] Vigueira CC, Li W, Olsen KMJ (2013). The role of Bh4 in parallel evolution of hull colour in domesticated and weedy rice. Evol Biol.

[40] Zhu BF, Si L, Wang Z, Zhou Y, Zhu J, Shangguan Y (2011). Genetic control of a transition from black to straw-white seed hull in rice domestication. Plant Physiol.

[41] Ahuja U, Ahuja SC, Chaudhary N, Thakrar R (2007). Red rices—past, present and future. Asian Agri Hist.

[42] Ray A, Deb D, Ray R, Chattopadhayay B (2013). Phenotypic characters of rice landraces reveal independent lineages of short-grain aromatic indica rice. AoB Plants.

[43] Siddiqui SU, Kumamaru T, Satoh H (2007). Pakistan rice genetic resources. I. Grain morphological diversity and its distribution. Pakistan J Bot.

[44] Kohli S, Mohapatra T, Das SR, Singh AK, Tandon V, Sharma RP (2004). Composite genetic structure of rice land races revealed by STMS markers. Curr Sci.

[45] Roy PS, Jena S, Maharana A, Rao GJN, Patnaik SSC (2014). Molecular characterization of short grain aromatic rice landraces of Odisha for detection of aroma. Oryza.

[46] Bisne R, Sarawgi AK (2008). Agro-morphological and quality characterization of Badshahbhog group from aromatic rice germplasm of Chhattisgarh. Bangladesh J Agril Res.

[47] Raghunathachari P, Khanna VK, Singh US (2000). RAPD analysis of genetic variability in Indian scented rice germplasm (*Oryza sativa* L.). Curr Sci.

[48] Reddy VS, Dash S, Reddy AR (1995). Anthocyanin pathway in rice (*Oryza sativa* L.): identification of a mutant showing dominant inhibition of anthocyanins in leaf and accumulation of proanthocyanidins in pericarp. Theor Appl Genet.

[49] Ramiah K (1953). Rice breeding and genetics. Science monograph. 19.

[50] Varaprasad GS, Shobha Rani N, Padmavati G, Sesu Madhav M, Bentur JS, Laksmi VJ (2013). Catalogue on aromatic short grain rices of India. DRR Technical Bulletin No. 69.

[51] Bajracharya J, Steele KA, Jarvis DI, Sthapit BR, Witcombe JR (2006). Rice landrace diversity in Nepal: variability of agro-morphological traits and SSR markers in landraces from a high-altitude site. Fields Crop Res.

[52] Dikshit N, Das AB, Sivaraj N, Kar MK (2013). Phenotypic diversity for agro-morphological traits in 105 landraces of rice (*Oryza sativa* L.) from Santhal Parganas, Jharkhand, India. Proc Natl Acad Sci India Sect B.

[53] Choudhury B, Khan ML, Dayanandan S (2013). Genetic structure and diversity of indigenous rice (*Oryza sativa*) varieties in the Eastern Himalayan region of Northeast India. Springer Plus.

[54] Tiwari KK, Singh A, Pattnaik S, Sandhu M, Kaur S, Jain S, Tiwari S (2015). Identification of a diverse mini-core panel of Indian rice germplasm based on genotyping using microsatellite markers. Plant Breed.

[55] Kim HJ, Jeong EG, Ahn SN, Doyle J, Singh N, Greenberg AJ, Won YJ, McCouch SR (2014). Nuclear and chloroplast diversity and phenotypic distribution of rice (*Oryza sativa* L.) germplasm from the democratic people’s republic of Korea. Rice.

[56] Laido G, Mangini G, Taranto F, Gadaleta A, Blanco A, Cattivelli L (2013). Genetic diversity and population structure of tetraploid wheats (*Triticum turgidum* L.) estimated by SSR, DArT and pedigree data. PLoS ONE.

[57] Yan W, Hu B, Zhang QJ, Jia L, Jackson A, Pan X, Huang B, Yan Z, Deren C. Short and erect rice (ser) mutant from Khao Dawk Mali 105 improves plant architecture. Plant Breed. 2012;131:282–5. doi:10.1111/j.1439-0523.2011.01943.x.

[58] Ookawa T, Yasuda K, Kato H, Sakai M, Seto M, Sunaga K, Motobayashi T, Tojo S, Hirasawa T (2010). Biomass production and lodging resistance in ‘Leaf Star’, a new long-culm rice forage cultivar. Plant Prod Sci.

[59] Ogunbayo SA, Si M, Ojo DK, Sanni KA, Akinwale MG, Toulou B, Shittu A, Idehen EO, Popoola AR, Daniel IO, Gregorio GB (2014). Genetic variation and heritability of yield and related traits in promising rice genotypes (*Oryza sativa* L.). J Plant Breed Crop Sci.

[60] Deb D (2005). Seeds of tradition, seeds of future, folk rice varieties of Eastern India.

[61] Rabara RC, Ferrer MC, Diaz CL, Newingham MCV, Romero GO (2014). Phenotypic diversity of farmers’ traditional rice varieties in the Philippines. Agronomy.

[62] Yamasaki M, Tenaillon MI, Bi IV, Schroeder SG, Sanchez-Villeda H, Doebley JF, Gaut BS, McMullen MD (2005). A large-scale screen for artificial selection in maize identifies candidate agronomic loci for domestication and crop improvement. Plant Cell.

[63] Samal KC, Rout GR, Das SR (2014). Study of genetic divergence of Indigenous Aromatic Rice (*Oryza Sativa* L.): potentials and consequences of on-farm management in traditional farming. J Agric Sci.

[64] Chase MW, Cowan RS, Hollingsworth PM, vanden Berg C, Madrinan S, Petersen G (2007). A proposal for a standardised protocol to barcode all land plants. Taxon.

[65] Pasqualone A, Lotti C, Blanco A (1999). Identification of durum wheat cultivars and monovarietal semolinas by analysis of DNA microsatellites. Eur Food Res Technol.

[66] Ren X, Zhu X, Warndorff M, Bucheli P, Shu Q (2006). DNA extraction and fingerprinting of commercial rice cereal products. Food Res Int.

[67] Salem HH, Ali BA, Huang TH, Qin DN, Wang XM, Xie QD (2007). Use of random amplified polymorphic DNA analysis for economically important food crops. J Integr PlANT Biol.

[68] Terzi V, Morcia C, Gorrini A, Stanca AM, Shewry PR, Faccioli P (2005). DNA-based methods for identification and quantification of small grain cereal mixtures and fingerprinting of varieties. J Cereal Sci.

[69] De Mattia F, Bruni I, Galimberti A, Cattaneo F, Casiraghi M, Labra M (2011). A comparative study of different DNA barcoding markers for the identification of some members of Lamiacaea. Food Res Int.

[70] Nicole S, Erickson DL, Ambrosi D, Bellucci E, Lucchin M, Papa R, Kress WJ, Barcaccia G, Donini P (2011). Biodiversity studies in Phaseolus species by DNA barcoding. Genome.

[71] Rao LVS, Prasad GS, Chiranjivi M, Chaitanya U, Surendhar R (2013). DUS characterization for farmer varieties of rice. IOSR J Agric Vet Sci.

[72] Panda D, Bisoi SS, Palita SK (2014). Floral diversity conservation through sacred groves in Koraput district, Odisha, India: a case study. Int Res J Environ Sci.

[73] Singh AK (2013). Probable agricultural biodiversity heritage sites in India: XVI. The Koraput region. Asian Agri Hist.

[74] Sharma SD, Tripathy S, Biswal J, Nanda JS (2000). Origin of *O. sativa* and its ecotypes. Rice breeding and genetics: research priorities and challenges.

[75] Fuller DQ (2006). Agricultural origins and frontiers in South Asia: a working synthesis. J World Prehist.

[76] Fuller DQ (2011). Finding plant domestication in the Indian subcontinent. Curr Anthropol.

[77] Choudhury PR, Kohli S, Srinivasan K, Mohapatra T, Sharma RP (2001). Identification and classification of aromatic rices based on DNA fingerprinting. Euphytica.

