# Supplementary material for: Differentiation and description of aromatic short grain rice landraces of eastern Indian state of Odisha based on qualitative phenotypic descriptors
Source: BMC Ecol. 2016 Aug 9;16:36. doi: 10.1186/s12898-016-0086-8 (PMC4977617; doi:10.1186/s12898-016-0086-8)
Supplement: Supplementary file 1 — 10.1186/s12898-016-0086-8 Aromatic short grain rice genotypes used in the present study and region of their collection. Table S2. Population statistics of the estimated clusters. *SP 1 and SP 2 are estimated subpopulations. Table S3. Pair-wise Nei’s unbiased genetic distance of 19 geographical districts. [file 12898_2016_86_MOESM1_ESM.docx]

**Table S1** Aromatic short grain rice genotypes used in the present study and region of their collection

| **Sl. No.** | **Accession number** | **Varieties** | **Area of collection** | **State** |
| --- | --- | --- | --- | --- |
| 1 | 44119 | Baluchi | Dhenkanal | Orissa |
| 2 | 44120 | Acharmati-1 | Bolangir | Orissa |
| 3 | 44121 | Acharmati-2 | Bolangir | Orissa |
| 4 | 44122 | Basaya bhog | Sundargarh | Orissa |
| 5 | 44123 | Basanasapuri | Puri | Orissa |
| 6 | 44124 | Basua bhog-1 | Anugul | Orissa |
| 7 | 44125 | Baukunja | Cuttack | Orissa |
| 8 | 44126 | Basasa phool | Bolangir | Orissa |
| 9 | 44127 | Badsabhog | Bolangir | Orissa |
| 10 | 44128 | Bhatagundi | Koraput | Orissa |
| 11 | 44129 | Bhadraka Basumati | Balasore | Orissa |
| 12 | 44130 | Basumati-1 | Cuttack | Orissa |
| 13 | 44131 | Baiganamanji | Bhawanipatna | Orissa |
| 14 | 44132 | Basaparijata | Kalahandi | Orissa |
| 15 | 44133 | Basanapuri | Puri | Orissa |
| 16 | 44134 | Basubhog | Koraput | Orissa |
| 17 | 44135 | Basanadhan | Koraput | Orissa |
| 18 | 44136 | Basanaphula | Cuttack | Orissa |
| 19 | 44137 | Chatianaki | Cuttack | Orissa |
| 20 | 44138 | Deulabhog-1 | Puri | Orissa |
| 21 | 44139 | Deulabhog-2 | Puri | Orissa |
| 22 | 44140 | Dhusara | Cuttack | Orissa |
| 23 | 44141 | Dubrajsena | Koraput | Orissa |
| 24 | 44142 | Durgabhog | Keonjhar | Orissa |
| 25 | 44143 | Dhurabahila | Koraput | Orissa |
| 26 | 44144 | Deulabhog-3 | Koraput | Orissa |
| 27 | 44145 | Dangar Basumati | Koraput | Orissa |
| 28 | 44146 | Dubraj | Koraput | Orissa |
| 29 | 44147 | Ganjamlocal-1 | Ganjam | Orissa |
| 30 | 44148 | Ganjamlocal-2 | Ganjam | Orissa |
| 31 | 44149 | Ganjeikali | Dhenkanal | Orissa |
| 32 | 44150 | Jaiphool | Bolangir | Orissa |
| 33 | 44151 | Jhillipanjar | Cuttack | Orissa |
| 34 | 44152 | Jala | Keonjhar | Orissa |
| 35 | 44153 | Jhingisali | Balasore | Orissa |
| 36 | 44154 | Kalajeera-1 | Mayurbhanj | Orissa |
| 37 | 44155 | Karpurkali | Ganjam | Orissa |
| 38 | 44156 | Kalikati-1 | Kalahandi | Orissa |
| 39 | 44157 | Kala krishna | Kalahandi | Orissa |
| 40 | 44158 | Kukudajata | Koraput | Orissa |
| 41 | 44159 | Koiamba-543 | Koraput | Orissa |
| 42 | 44160 | Kanakchampa | Keonjhar | Orissa |
| 43 | 44161 | Karpurabasa | Koraput | Orissa |
| 44 | 44162 | Krishnabhog | Puri | Orissa |
| 45 | 44163 | Kalajiri-1 | Puri | Orissa |
| 46 | 44164 | Karpurazeera | Kalahandi | Orissa |
| 47 | 44165 | Kendumanjee | Koraput | Orissa |
| 48 | 44166 | Laxmibilas-1 | Deogarh | Orissa |
| 49 | 44167 | Laxmibilas-2 | Sambalpur | Orissa |
| 50 | 44168 | Leelabati | Balasore | Orissa |
| 51 | 44169 | Lektimachi-1 | Malkangiri | Orissa |
| 52 | 44170 | Lektimasi | Malkangiri | Orissa |
| 53 | 44171 | Lektimachi-2 | Malkangiri | Orissa |
| 54 | 44172 | Laser | Malkangiri | Orissa |
| 55 | 44173 | Mahulakuchi | Malkangiri | Orissa |
| 56 | 44174 | Magura selectioin | Ganjam | Orissa |
| 57 | 44175 | Manas | Puri | Orissa |
| 58 | 44176 | Manasi | Puri | Orissa |
| 59 | 44177 | Mahulkuchi | Malkangiri | Orissa |
| 60 | 44178 | Nalidhan | Cuttack | Orissa |
| 61 | 44179 | Nanu | Anugul | Orissa |
| 62 | 44180 | Pirima | Koraput | Orissa |
| 63 | 44181 | Panasmanjee | Malkangiri | Orissa |
| 64 | 44182 | Sunsuniasunaphul | Deogarh | Orissa |
| 65 | 44183 | Badaguda | Deogarh | Orissa |
| 66 | 44184 | Benugopal | Sambalpur | Orissa |
| 67 | 44185 | Jayaphul | Sundargarh | Orissa |
| 68 | 44186 | Benubhog | Mayurbhanj | Orissa |
| 69 | 44187 | Samleibhog-1 | Sundargarh | Orissa |
| 70 | 44188 | Bhuinsasal | Deogarh | Orissa |
| 71 | 44189 | Kalajira | Dhenkanal | Orissa |
| 72 | 44190 | Laxmikajol | Keonjhar | Orissa |
| 73 | 44191 | Shantibhog | Puri | Orissa |
| 74 | 44192 | Sujata | Puri | Orissa |
| 75 | 44193 | Thakursuna | Cuttack | Orissa |
| 76 | 44194 | Suman | Cuttack | Orissa |
| 77 | 44195 | Thakur bhog | Puri | Orissa |
| 78 | 44196 | Atmasital-1 | Koraput | Orissa |
| 79 | 44197 | Nagri | Koraput | Orissa |
| 80 | 44198 | Pipalbasa | Sambalpur | Orissa |
| 81 | 44199 | Samleibhog-2 | Sundargarh | Orissa |
| 82 | 44200 | Kalazeera | Dhenkanal | Orissa |
| 83 | 44201 | Laxmibilas-3 | Sambalpur | Orissa |
| 84 | 44202 | Basnadhan-1 | Sundargarh | Orissa |
| 85 | 44203 | Kalaziri | Ganjam | Orissa |
| 86 | 44204 | Basumati-2 | Sundargarh | Orissa |
| 87 | 44205 | Parijatak | Ganjam | Orissa |
| 88 | 44206 | Magura | Ganjam | Orissa |
| 89 | 44207 | Gadakakudinga | Phulbani | Orissa |
| 90 | 44208 | Gangabali | Ganjam | Orissa |
| 91 | 44209 | Karpurakranti | Ganjam | Orissa |
| 92 | 44210 | Phulabani local | Phulbani | Orissa |
| 93 | 44211 | Kalajeera-2 | Ganjam | Orissa |
| 94 | 44212 | Kalagiri | Cuttack | Orissa |
| 95 | 44213 | Nadiarasa | Cuttack | Orissa |
| 96 | 44214 | Kendragali | Cuttack | Orissa |
| 97 | 44215 | Saragadhuli | Cuttack | Orissa |
| 98 | 44216 | Karpurakanta | Cuttack | Orissa |
| 99 | 44217 | Basumati-3 | Kendrapara | Orissa |
| 100 | 44218 | Basuabhog-2 | Kendrapara | Orissa |
| 101 | 44219 | Garmatia | Puri | Orissa |
| 102 | 44220 | Krisna bhog | Puri | Orissa |
| 103 | 44221 | Tulasi basa | Nayagarh | Orissa |
| 104 | 44222 | Kalatulasi | Nayagarh | Orissa |
| 105 | 44223 | Kalajeera-3 | Nayagarh | Orissa |
| 106 | 44224 | Batakarua | Keonjhar | Orissa |
| 107 | 44225 | Basumati-4 | Jajpur | Orissa |
| 108 | 44226 | Kalajiri-2 | Ganjam | Orissa |
| 109 | 44227 | Suetpotato | Jajpur | Orissa |
| 110 | 44228 | Maharaji | Kalahandi | Orissa |
| 111 | 44229 | Laktimachi | Koraput | Orissa |
| 112 | 44230 | Karpurakali | Ganjam | Orissa |
| 113 | 44231 | Pimpudibasa | Mayurbhanj | Orissa |
| 114 | 44232 | Atmasital-2 | Malkangiri | Orissa |
| 115 | 44233 | Kalajeera-4 | Koraput | Orissa |
| 116 | 44234 | Nadiaphool | Cuttack | Orissa |
| 117 | 44235 | Jawaphool | Bolangir | Orissa |
| 118 | 44236 | Kalikati-2 | Bhawanipatna | Orissa |
| 119 | 44237 | Basnadhan-2 | Bhawanipatna | Orissa |
| 120 | 44238 | Morllu | Bhawanipatna | Orissa |
| 121 | 44239 | Basanaparijata | Bhawanipatna | Orissa |
| 122 | 44240 | Lilabati | Balasore | Orissa |
| 123 | 44241 | Ramabana Basmati | Bolangir | Orissa |
| 124 | 44242 | Kalkati | Bolangir | Orissa |
| 125 | 44243 | Nadiakata | Bolangir | Orissa |
| 126 | 44244 | Kalakanhu | Bolangir | Orissa |

**Table S2** Population statistics of the estimated clusters

| Population* | Membership (%) | *F*_ST_ | Average distances | Allele frequency divergence |
| --- | --- | --- | --- | --- |
| *SP 1* (8 admixtures) | 54.8 | 0.1415 | 0.2529 | 0.0489 |
| *SP 2* (7 admixtures) | 45.2 | 0.2794 | 0.2765 |  |

**SP 1* and *SP 2* are estimated subpopulations

**Table S3** Pair-wise Nei’s unbiased genetic distance of 19 geographical districts

| **Districts** | **1** | **2** | **3** | **4** | **5** | **6** | **7** | **8** | **9** | **10** | **11** | **12** | **13** | **14** | **15** | **16** | **17** | **18** | **19** |
| --- | --- | --- | --- | --- | --- | --- | --- | --- | --- | --- | --- | --- | --- | --- | --- | --- | --- | --- | --- |
| **Anugul** | **** |  |  |  |  |  |  |  |  |  |  |  |  |  |  |  |  |  |  |
| **Balasore** | 0.163 | **** |  |  |  |  |  |  |  |  |  |  |  |  |  |  |  |  |  |
| **Balangir** | 0.103 | 0.055 | **** |  |  |  |  |  |  |  |  |  |  |  |  |  |  |  |  |
| **Cuttack** | 0.103 | 0.044 | 0.026 | **** |  |  |  |  |  |  |  |  |  |  |  |  |  |  |  |
| **Deogarh** | 0.172 | 0.103 | 0.074 | 0.063 | **** |  |  |  |  |  |  |  |  |  |  |  |  |  |  |
| **Dhenkanal** | 0.223 | 0.112 | 0.094 | 0.082 | 0.108 | **** |  |  |  |  |  |  |  |  |  |  |  |  |  |
| **Ganjam** | 0.068 | 0.081 | 0.035 | 0.015 | 0.073 | 0.104 | **** |  |  |  |  |  |  |  |  |  |  |  |  |
| **Jajpur** | 0.148 | 0.145 | 0.114 | 0.089 | 0.146 | 0.196 | 0.068 | **** |  |  |  |  |  |  |  |  |  |  |  |
| **Kalahandi** | 0.098 | 0.041 | 0.024 | 0.013 | 0.051 | 0.078 | 0.025 | 0.108 | **** |  |  |  |  |  |  |  |  |  |  |
| **Kendrapara** | 0.165 | 0.114 | 0.158 | 0.103 | 0.144 | 0.172 | 0.121 | 0.241 | 0.084 | **** |  |  |  |  |  |  |  |  |  |
| **Keonjhar** | 0.102 | 0.073 | 0.042 | 0.035 | 0.096 | 0.105 | 0.032 | 0.108 | 0.030 | 0.141 | **** |  |  |  |  |  |  |  |  |
| **Koraput** | 0.104 | 0.045 | 0.024 | 0.028 | 0.067 | 0.081 | 0.042 | 0.106 | 0.031 | 0.126 | 0.042 | **** |  |  |  |  |  |  |  |
| **Malkangiri** | 0.093 | 0.060 | 0.016 | 0.020 | 0.073 | 0.106 | 0.027 | 0.112 | 0.024 | 0.135 | 0.035 | 0.027 | **** |  |  |  |  |  |  |
| **Mayurbhanj** | 0.164 | 0.099 | 0.101 | 0.081 | 0.167 | 0.113 | 0.104 | 0.133 | 0.084 | 0.199 | 0.112 | 0.068 | 0.097 | **** |  |  |  |  |  |
| **Nayagarh** | 0.184 | 0.113 | 0.077 | 0.077 | 0.131 | 0.133 | 0.099 | 0.222 | 0.067 | 0.188 | 0.102 | 0.104 | 0.083 | 0.126 | **** |  |  |  |  |
| **Phulbani** | 0.232 | 0.189 | 0.119 | 0.127 | 0.182 | 0.204 | 0.107 | 0.137 | 0.151 | 0.370 | 0.108 | 0.139 | 0.130 | 0.223 | 0.202 | **** |  |  |  |
| **Puri** | 0.115 | 0.042 | 0.041 | 0.021 | 0.090 | 0.098 | 0.036 | 0.106 | 0.032 | 0.098 | 0.051 | 0.029 | 0.047 | 0.077 | 0.097 | 0.150 | **** |  |  |
| **Sambalpur** | 0.164 | 0.074 | 0.055 | 0.049 | 0.086 | 0.087 | 0.057 | 0.124 | 0.033 | 0.122 | 0.066 | 0.073 | 0.068 | 0.131 | 0.085 | 0.183 | 0.062 | **** |  |
| **Sundargarh** | 0.111 | 0.081 | 0.047 | 0.024 | 0.080 | 0.106 | 0.029 | 0.100 | 0.036 | 0.138 | 0.038 | 0.038 | 0.033 | 0.079 | 0.102 | 0.138 | 0.033 | 0.078 | **** |
