# Supplementary material for: Differentiation and description of aromatic short grain rice landraces of eastern Indian state of Odisha based on qualitative phenotypic descriptors
Source: BMC Ecol. 2016 Aug 9;16:36. doi: 10.1186/s12898-016-0086-8 (PMC4977617; doi:10.1186/s12898-016-0086-8)
Supplement: Supplementary file 2 — 10.1186/s12898-016-0086-8 Sampling sites in nineteen districts of Odisha (India) and information on population structure. Figure S2. Frequency distribution of phenotypic traits among 126 short grain aromatic rice landraces. Figure S3. The relationship between ΔK and K showing the highest peak at K = 2. [file 12898_2016_86_MOESM2_ESM.docx]

**Figure S1** Sampling sites in nineteen districts of Odisha (India) and information on population structure


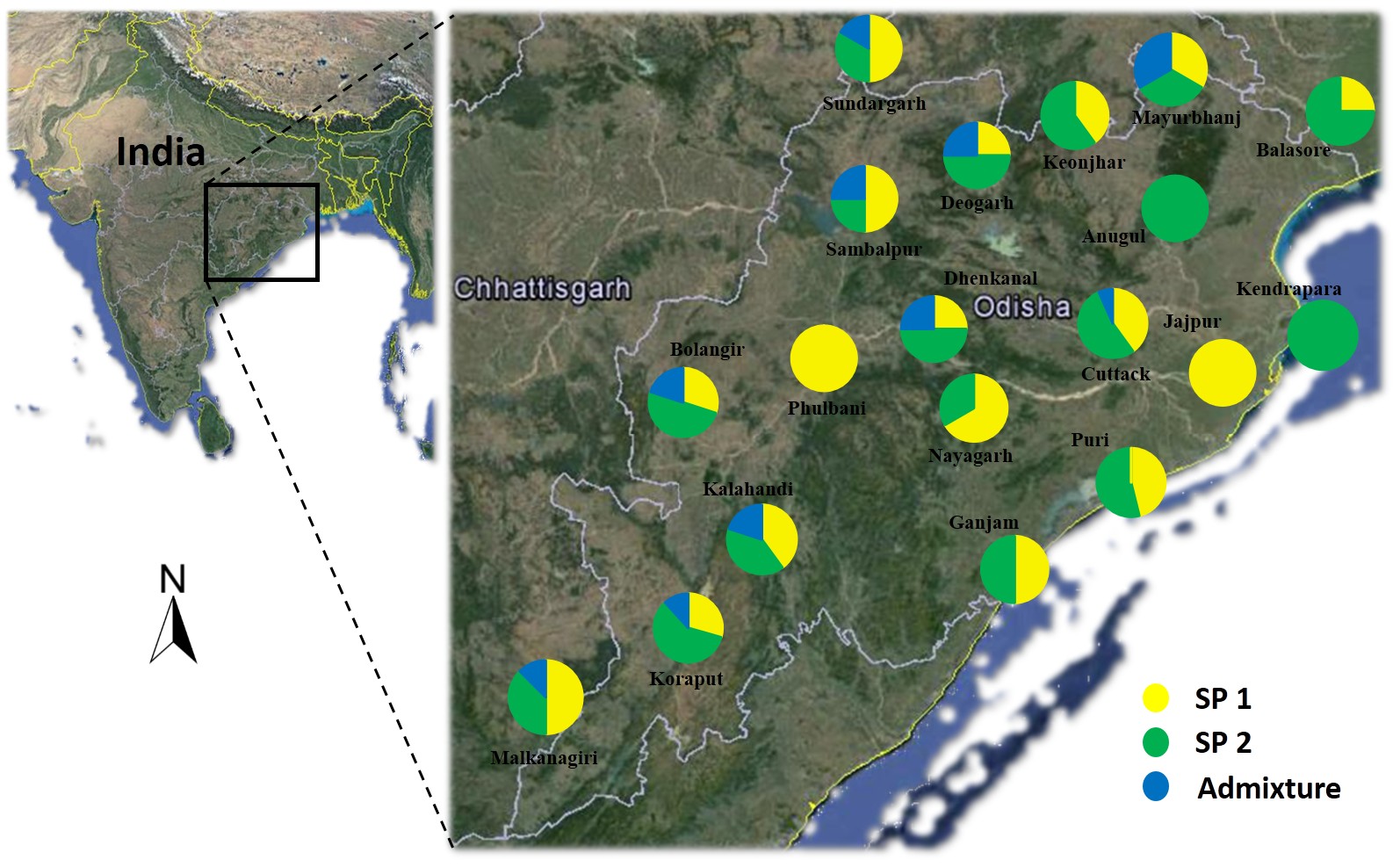


**Figure S2** Frequency distribution of phenotypic traits among 126 short grain aromatic rice landraces


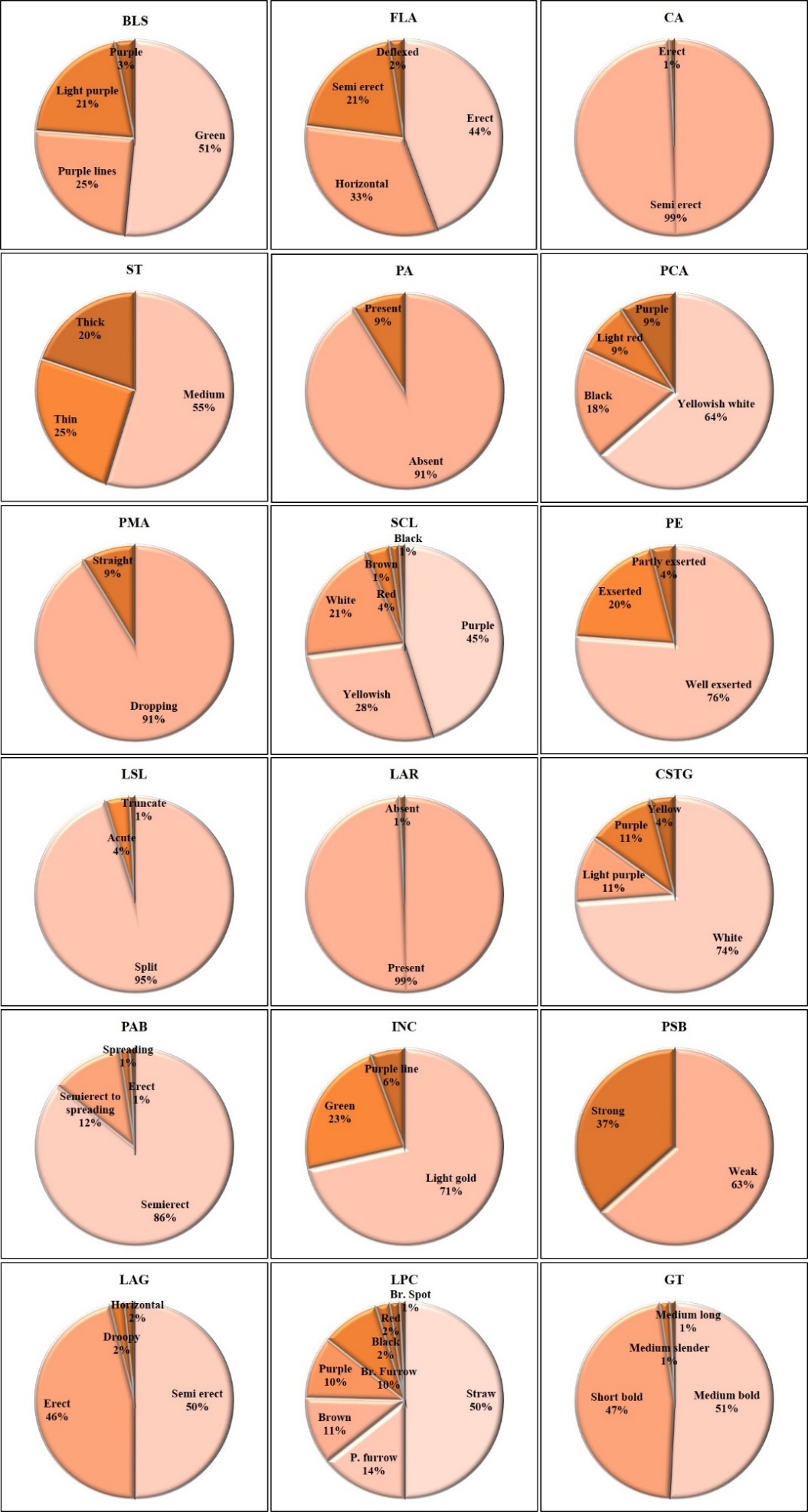


**Figure S3** The relationship between ΔK and K showing the highest peak at K = 2.
